# TidyGWAS: a scalable approach for standardized cleaning of genome-wide association study summary statistics

**DOI:** 10.1093/bioadv/vbaf262

**Published:** 2025-10-27

**Authors:** Arvid Harder, Jerry Guintivano, Joëlle A Pasman, Patrick F Sullivan, Yi Lu

**Affiliations:** Department of Medical Epidemiology and Biostatistics, Karolinska Institutet, Stockholm, 171 77, Sweden; Department of Genetics and Psychiatry, University of North Carolina, Chapel Hill, NC, NC 27599-7264, United States; Genetic Epidemiology, Department of Psychiatry, Amsterdam UMC Location University of Amsterdam, Amsterdam, 1105 AZ, The Netherlands; Department of Medical Epidemiology and Biostatistics, Karolinska Institutet, Stockholm, 171 77, Sweden; Genetic Epidemiology, Department of Psychiatry, Amsterdam UMC Location University of Amsterdam, Amsterdam, 1105 AZ, The Netherlands; Department of Medical Epidemiology and Biostatistics, Karolinska Institutet, Stockholm, 171 77, Sweden

## Abstract

**Motivation:**

Genome-wide association studies (GWAS) have transformed human genetics by identifying tens of thousands of trait-associated variants, enabling applications from drug discovery to polygenic risk prediction. These advancements depend critically on open sharing of GWAS summary statistics. However, a lack of standardized formats complicates downstream analyses, requiring extensive dataset-specific “munging” before analysis can proceed.

**Results:**

Here we present tidyGWAS, an R package that streamlines this process by cleanly separating data validation and harmonization from quality control. tidyGWAS uses curated data to repair and harmonize variant identifiers across genome builds, imputes missing columns when possible, and validates summary statistics with minimal filters. Outputs are saved as partitioned parquet files, optimized for high-throughput analysis via the arrow package. Benchmarked against existing tools tidyGWAS is up to 6.5× faster and substantially more memory efficient. Additionally, we implement a fixed-effects meta-analysis directly on tidyGWAS output, achieving up to 10× speedup over existing software. tidyGWAS simplifies and accelerates statistical genetic workflows, improving reproducibility and scalability for large-scale genetic analyses.

**Availability and implementation:**

The package, reference data, and Docker containers are freely available for broad adoption.

## 1 Introduction

The advent of microarray-based genotyping has enabled genome-wide association studies (GWAS), which have been remarkably successful in detecting robust and replicable associations between human phenotypes and genotypes. At the end of 2021, the NHGRI/EBML GWAS catalog documented 4593 studies covering 3908 traits, revealing 107 685 unique SNPs significantly associated with traits at a stringent GWAS *P* value threshold (Sullivan *et al.* 2023). These associations have benefited drug discovery, as medicines supported by evidence from human genetics are estimated to have a 2.6-fold higher likelihood of progressing from clinical development to approval (Minikel *et al.* 2020).

A notable aspect of the genetics research community has been its early adoption of open science principles that makes GWAS summary statistics freely available without compromising data privacy (Uffelman et al. 2021). This has enabled the development of a wide range of downstream algorithms and methods based on GWAS summary statistics, such as credible set analysis (Zou *et al.* 2022), polygenic scores (Zheng *et al.* 2024), gene-based tests (de Leeuw *et al.* 2015), functional assays (McAfee *et al.* 2023), identification of critical tissues and cell types (Finucane *et al.* 2015, Zhang *et al.* 2022), and estimation of SNP-based heritability and shared genetic covariance between traits (Bulik-Sullivan *et al.* 2015). Such advancements would not have been possible without the sharing of summary statistics.

However, there is currently no widely agreed-upon standard for sharing summary statistics. Efforts are underway to establish such standards, including initiatives to create unified data formats and sharing protocols (MacArthur *et al.* 2021). The diversity in data formats extends to secondary algorithms and methods, which frequently necessitate the input of summary statistics with a broad array of filters, column names, and data structures. This has made “munging” the initial step in any analysis using summary statistics. Munging broadly involves three types of tasks: (i) data validation (e.g. removing rows with allele frequencies or *P* values outside the 0–1 range), (ii) data harmonization (representing both “chr1,” “ch1,” and “1” as chromosome 1, or acquiring rsID from chromosome and position, or adding allele frequencies from a reference dataset), and (iii) data quality control (such as removing ambiguous SNPs, removing variants with low imputation accuracy or restricting to variants with minor allele frequency >1%). With the growing availability of summary statistics for various human traits, diseases, and disorders, it is not uncommon to analyze hundreds of traits to answer a research question (Zheng *et al.* 2017). Munging can be time-consuming when processing a large set of summary statistics, since each dataset may present unique challenges. Moreover, employing multiple secondary methods often requires several rounds of munging, as each software package typically requires a different input format and quality control.

Several methods have been developed to tackle this problem, such as MungeSumstats (Murphy *et al.* 2021), Cleansumstats (Gadin *et al.* 2022), or SumstatsRehab (Matushyn *et al.* 2022). However, these methods typically combine harmonization, validation, and quality control. When using multiple downstream applications, this often results in having to execute the software repeatedly, with a new set of parameters for each downstream application. For example, the removal of “strand-ambiguous” SNPs is mandatory in Cleansumstats and is standard for Mendelian randomization analysis or polygenic scores but not when selecting variants for functional assays.

Here we sought to cleanly delineate between quality control, harmonization, and validation. We developed the R package tidyGWAS as an alternative to existing software. tidyGWAS is entirely focused on data validation and harmonization, applying only a set of minimal filters. tidyGWAS returns information about variant identity from both GRCh37, GRCh38 and imputes missing columns if possible. tidyGWAS outputs a cleaned format that can be continuously reused in most downstream analysis and provides the user with the necessary data to flexibly match the output format with external software.

## 2 Methods

tidyGWAS is implemented in R (version 4.4) and allows users to clean and “munge” GWAS summary statistics within the R environment. The full procedure of tidyGWAS is displayed in Table 1. To perform validation and reparation of variant identity (chromosome and position, rsID, effect allele, and other allele), we used dbSNP (v.155) without indels as the reference. We applied the following filters to the reference data.

**Table 1.** The tidyGWAS algorithm.

Step	Action	Function	Explanation
1	Parse the input format	parse_input()	tidyGWAS accepts four types of input for the main argument “tbl”: a GWAS catalog study id: “GSCT000001” a URL an in-memory data.frame a file path In the case of (1) and (2), tidyGWAS will attempt to download the file and execute the algorithm on the downloaded file.
2	Parse the column names of the file and map to the tidyGWAS columns. drop incorrect columns or columns with all missing	guess_names(), select_correct_columns()	tidyGWAS will attempt to guess the correct column name mapping. Columns that cannot be mapped to a tidyGWAS column are dropped. Column names can manually be set using ′column_names′ if names are guessed incorrectly.
3	Remove rows with missing values in critical columns	remove_rows_with_na()	EffectAllele and OtherAllele are always required. Either RSID or CHR and POS are additionally required. Rows with missing values in these columns are dropped. If all of CHR, POS and RSID are present, RSID is dropped (and subsequently repaired using reference data).
4	Remove rows with duplicated variant id	remove_duplicates()	Deduplicates rows using either CHR: POS: EffectAllele: OtherAllele or RSID: EffectAllele: OtherAllele.
5	Detect and process indels separately	detect_indels()	Indels are detected by examining the character length of the effect allele and other allele columns (>1 character) is coded as indel. The characters “D,” “I,” and “R” are also coded as indels. If indel_strategy = “qc,” indels will be matched to the dbSNP reference data.
6	Validation of a subset of columns	validate_columns()	Columns are coerced to the correct type and a set of column validation checks are applied (Table 1, available as supplementary data at *Bioinformatics Advances* online). Rows that fail validation checks are removed.
7	Imputation/repair of RSID or CHR and POS Remove variants without a dbSNP entry, and variants with alleles incompatible with the dbSNP reference alleles	repair_ids()	There are three valid combinations of input columns to represent variant identity: 1. Chromosome and position exists, but not RSID 2. Chromosome and position and RSID exists 3. RSID exists, but not chromosome and position. Option (1) and (2) are considered identical by tidyGWAS, as in the case of (2) it drops the RSID column and returns the RSID from dbSNP. For option (3), RSIDs are updated with the list of merged SNPs from dbSNP. For rows with CHR: POS: REF: ALT format, the values are split to get CHR and POS. The summary statistics merge with the dbSNP reference files either using CHR: POS (1 and 2) or RSID (3). Variants without a dbSNP entry or variants with alleles that are not compatible with dbSNP reference and alternate alleles are dropped.
8	Identify multiallelic variants	flag_duplicates()	multi_allelic is defined by the presence of multiple identical RSIDs.
9	Repair missing statistics column: Z, B, SE, P if possible.	repair_stats()	With assumptions, important columns can be calculated based on other columns. **Z from B and SE** Z = B/SE **Z from B and P** sign(B) × sqrt(stats::qchisq(P, 1, lower=FALSE)) **B from Z, N, EAF** Z/sqrt((2 × EAF × (1 − EAF)) × (N + (Z^2))) **SE from Z, N, EAF** 1/sqrt((2 × EAF) × (1 − (EAF)) × (N + (Z^2))) **P from Z** stats::pnorm(−abs(Z)) × 2 **N from SE and MAF** 4/((2 × MAF × (1 − MAF)) × SE^2)
10	Estimate ancestry composition (optional)	ancestry_comp()	Estimate ancestry composition using the allele frequency estimates from the summary statistics. Algorithm and reference data from Privé, 2022.
11	Detect allele frequency mismatches	add_freq_diff_flag()	Flag variants with allele frequence >0.2 compared to chosen reference. Users can specify one of five superpopulations from 1000G reference data.
**Downstream analysis in tidyGWAS**			
**Action**		**Function**	**Explanation**
Meta analyze a set of tidyGWASsed summary statistics		meta_analyse()	Apply fixed-effects meta-analysis across a set of summary statistics cleaned with tidyGWAS. Requires output_format = “hivestyle” in tidyGWAS()
Estimate heritability, genetic correlations and partitioned heritability		from_tidyGWAS() munge() ldsc_h2() ldsc_rg() partition_h2()	Run LDscore regression directly inside R, using the output from tidyGWAS. Estimation requires reading in the file with “from_tidyGWAS()” “ldsc_h2()” and “ldsc_rg()” estimate heritability and genetic correlations. Example code: from_tidyGWAS(tidyGWAS_output) |> ldsc_h2()

1. removed entries where a RSID maps to multiple positions
2. from sets of entries with identical chromosome and position we selected the entry with the smallest rsID and removed the rest
3. removed entries where the chromosome varied between GRCh37 and GRCh38
4. kept entries with positions on both GRCh37 and GRCh38

We additionally provide an indel-specific reference dataset, based on dbSNP157, with left-aligned and trimmed allele representations. The curated dbSNP data are stored in parquet files and made publicly available at Zenodo. Matching to the curated dbSNP data uses the *arrow* package, optimizing performance and memory. The minimum set of columns required by tidyGWAS are those needed to determine variant identity: either chromosome and position, or rsID. Columns with effect allele and other allele are always required. Entries with missing values in mandatory columns are removed as well as entries with duplicated variant identity. Before matching with dbSNP, all columns are validated and harmonized, e.g. detecting entries with CHR: POS: REF: ALT in the rsID column, or the use of “chr1” instead of “1” in the chromosome column. The harmonization and validation of each column is detailed in Table 1, available as supplementary data at *Bioinformatics Advances* online, and the possible columns in the output file is detailed in Table 2, available as supplementary data at *Bioinformatics Advances* online. tidyGWAS merges input summary statistics with the dbSNP reference by matching on chromosome and position first; if either is missing, it uses rsID. Genome build is determined by matching 10 000 rows of the summary statistics to both builds and selecting the one for which most rows had a match after merging. Information on both genome builds is returned. tidyGWAS will attempt to impute missing columns from existing ones, e.g. calculating beta and standard errors from Z-score, sample size, and allele frequency. If allele frequency is missing, an optional argument allows for users to append precomputed allele frequency estimates from a chosen ancestry group from the 1000 Genomes dataset (Byrska-Bishop *et al.* 2022). Due to the large gain in computational efficiency, tidyGWAS outputs partitioned *parquet* files by default. This can be changed to a normal tab-separated file with the “output_format” argument.

## 3 Results

The main functionality of tidyGWAS is implemented in the tidyGWAS::tidyGWAS() function, which requires two mandatory arguments. The first is “tbl,” which accepts either a GWAS catalog id (“GSCT000000”), an in-memory R data.frame, a URL (“https://figshare.com/ndownloader/files/52419701”), or a local file path. If passed a URL or a GWAS catalog id, tidyGWAS will attempt to download the file and then execute the tidyGWAS::tidyGWAS() function. The second mandatory argument is “dbsnp_path,” a file path to the reference data folder, which users need to download prior to running tidyGWAS. Files are parsed using data.table::fread and tidyGWAS will attempt to automatically infer column names. Optional arguments exist to allow for duplicated variants, estimating ancestry proportions (Privé 2022) and flagging allele frequency differences with a chosen reference population. A docker container with tidyGWAS installed is made available through continuous integration at Dockerhub, allowing tidyGWAS to be easily implemented on high-performance clusters through the Singularity, Apptainer, or Docker softwares.

### 3.1 Benchmarking of tidyGWAS

**Figure 1.**
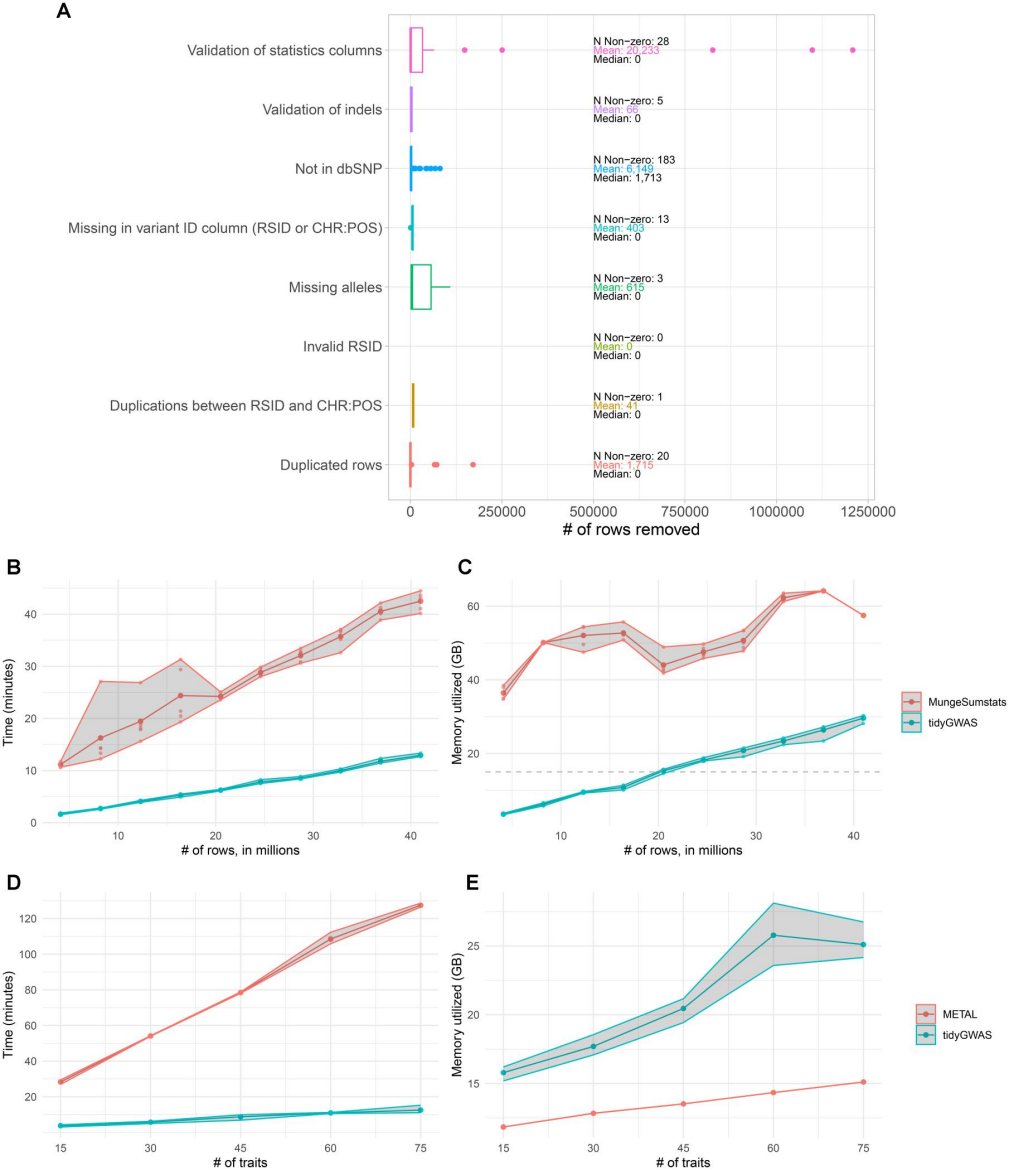
Benchmarking of tidyGWAS. (A) Boxplot of number of rows removed for each filter applied in tidyGWAS. The text adds additional information: number of summary statistics with more than 0 rows removed, the mean and median of rows removed. (B, C) Wall-clock time and memory utilized, from a benchmarking of tidyGWAS and MungeSumstats, across a number of rows: 4, 8, 12, 16, 20, 24, 28, 32, 36, 40 million. (D, E) Wall-clock time and memory utilized by tidyGWAS and METAL, when performing fixed-effects meta-analysis across a range summary statistics: 15, 30, 45, 60, 75.

We benchmarked the main tidyGWAS function against the main MungeSumstats function, [tidyGWAS::tidyGWAS(), MungeSumstats::format_sumstats()], using a large summary statistics file for eosinophil count (Vuckovic *et al.* 2020), downsampled to 4, 8, 12, 16, 20, 24, 28, 32, 36, and 40 million rows (Fig. 1B and C). Both functions align to a version of dbSNP as reference data and perform checks on columns. tidyGWAS was ∼6× faster compared to MungeSumstats on 8 million rows (mean execution time: 2.71 minutes vs. 16.32 minutes) and was more conservative in terms of memory usage (mean memory used: 6.2 GB vs. 50.1 GB). For the summary statistics with 40 million rows, tidyGWAS continued to perform better, although the relative difference was reduced (mean execution time: 12.9 minutes vs. 42.5 minutes, mean memory usage: 29.6 GB vs. 57.5 GB).

### 3.2 Applications of tidyGWAS

#### 3.2.1 Harmonizing 184 summary statistics

We applied tidyGWAS to a set of 184 unique GWAS summary statistics to investigate potential reasons for variant rows failing the basic quality control implemented in tidyGWAS. The most common reason for a row to be removed was not having a match with the dbSNP reference data (183 out of 184 summary statistics). For the remaining criteria, the modal number of rows removed were 0, meaning that most filters tended to not remove rows.

About 15.2% (28/184) of summary statistics had rows removed at the statistics validation step (e.g. missing effect size or standard error), 10.9% (20/184) had rows filtered due to duplications, and 7.0% (13/184) had rows missing variant ID. Other filters had limited impact on most summary statistics (>97%). Nonetheless, there are some notable outliers, e.g. in validation of statistics, and duplicates, where a subset of summary statistics had 25 000–1 000 000 rows removed (Fig. 1A).

#### 3.2.2 Multifile datasets analysis with the *arrow* package

The default output format of tidyGWAS is hivestyle-partitioned *parquet* files (by chromosome). The arrow package in R allows users to query and analyze large, distributed, larger-than-memory datasets that are stored as partitioned parquet files with minimal modifications to traditional data-handling code. Using the *arrow* package, multiple summary statistics can easily be analyzed as a single dataset. For example, querying for all significant variants across 184 summary statistics within a specified region (chr7:1788081–2289862, hg38, *MAD1L1*), is executed in ∼2 seconds (mean execution time: 1.82 seconds, 10 replicates) on our computational cluster (using 8 cores from an AMD EPYC 7742 64-Core Processor). This makes tasks such as intersecting specific variants and identifying top variants in a region across multiple traits significantly easier.

#### 3.2.3 Downstream applications

Downstream analyses of GWAS summary statistics. such as meta-analysis of contributing cohorts and the estimation of SNP-based heritability and genetic correlations, typically involve time-consuming formatting and data munging, and require switching to command-line applications such as METAL (Willer *et al.* 2010) or LDSC (Bulik-Sullivan et al. 2015). To improve the efficiency of overall analytical pipelines, we directly implemented a fixed-effects meta-analysis function in R for summary statistics, using the default tidyGWAS output format (partitioned parquet files). Our implementation was up to 10× faster than METAL (using the “analyze HETEROGENEITY” command, 75 summary statistics, mean execution time, 12.5 minutes vs. 127 minutes, mean memory usage 25.1 GB vs. 15 GB.1, three replicates, Fig. 1D and E). Importantly, the tidyGWAS implementation requires no munging and streamlines the process within a R session.

LDSC is a popular summary statistics-based tool to estimate SNP-based heritability and genetic correlations. However, the software package was written in Python 2.7.2, which requires upgrade and maintenance. To further streamline basic tasks, we implemented LDscore regression in R. We provide an interface to directly estimate heritability and genetic correlations requiring only the output of tidyGWAS as input, removing the previously cumbersome steps of writing out intermediate files to disk and using LDSC from the command-line interface. The functions are implemented in the ldsR package.

## 4 Conclusion

In the analysis of genetic data, considerable time is spent “munging” summary statistics, a process that is prone to human error. Downstream methods typically require different input formats, necessitating users to continually reformat data for various tasks. Here, we introduce tidyGWAS, designed to provide a harmonized summary statistics format as the foundational step for any downstream analysis. By separating data validation and harmonization from filtering, users can create standardized pipelines to reuse the same summary statistics file for multiple secondary analyses. Importantly, the design choices behind tidyGWAS aim to have it be executed only once—subsequent filters can then be applied for specific purposes, and the cleaned summary statistics can be reused continuously for different applications.

Summary statistics are typically stored as compressed text files. While this format is user-friendly, it is not optimized for computational efficiency. We demonstrate how the *parquet* format can significantly simplify analysis of multiple summary statistics. The partitioned format underpins our implementation of meta-analysis, offering faster processing compared to METAL, and significantly improving usability by making it accessible within the R environment without requiring further data harmonization.

In conclusion, tidyGWAS streamlines summary statistics handling by providing a unified format after validation and harmonization and simplifies reuse across downstream analyses. Separating cleaning from filtering reduces redundancy and enhances reproducibility. With a standard format, automating connections to downstream analysis is greatly simplified. By adopting the parquet format, tidyGWAS ensures faster processing, scalable meta-analyses, and seamless integration within R.

## Supplementary Material

vbaf262_Supplementary_Data

## Data Availability

The source code for tidyGWAS and LDSC (“ldsR”) in R is available at github. https://github.com/Ararder/tidyGWAS, https://github.com/Ararder/ldsR. Code used to generate benchmarks and figures for this paper: https://github.com/Ararder/tidyGWAS-code. Reference data for tidyGWAS https://zenodo.org/records/16639374. Docker container for tidyGWAS https://hub.docker.com/r/arvhar/tidygwas. Using docker or apptainer from the command line: docker run arvhar/tidygwas: latest, apptainer pull https://docker/arvhar/tidygwas:latest.
