# Supplementary material for: TidyGWAS: a scalable approach for standardized cleaning of genome-wide association study summary statistics
Source: Bioinform Adv. 2025 Oct 27;5(1):vbaf262. doi: 10.1093/bioadv/vbaf262 (PMC12597892; doi:10.1093/bioadv/vbaf262)
Supplement: vbaf262_Supplementary_Data [file vbaf262_supplementary_data.docx]

### Supplementary Table 1 – Column-wise quality control

| Column | Description |
| --- | --- |
| RSID | Coerced to character. A regex is used to detect values not corresponding to “rsXXX..”. Values not corresponding to a valid rsid are scanned for the presence of “CHR:POS:REF:ALT” using several different separators |
| CHR | Coerced to character and uppercase. Values in the format of “chr2” or “ch2” are converted to “2”.  “23” is converted to “X”.  “24” is converted to “Y”,  “25” is converted to “XY”,  “26” is converted to “MT”  “M” is converted to “MT”.  Values not in [1:22, “X”, “Y”, “MT”, “XY”] are removed |
| EffectAllele, OtherAllele | Coerced to character and converted to uppercase. Values not in [A,C,G,T] are removed. Indels handled separately |
| POS | Coerced to integer, NA/NAN/INF removed, values over 10^9 are removed |
| B | Coerced to double. NA/NAN/INF removed. User warned if median of B is between 0.9 and 1.1 (likely mislabelled odds ratio), or if the absolute median is over 0.1. |
| P | Coerced to double. Rows where P = 0 are converted to the user supplied value. NA/NAN/INF removed. Values outside [0, 1] are removed. |
| EAF | Coerced to double. NA/NAN/INF removed. Values outside [0,1] are removed |
| Z | Coerced to double. NA/NAN/INF removed |
| SE | Coerced to double. NA/NAN/INF removed. SE <= 0 removed. |
| N, CaseN, ControlN, EffectiveN | Coerced to integer. NA/NAN/INF removed. N <= 0 removed. |
| INFO | Coerced to double. NA/NAN/INF removed. Values outside of INFO [0, 2) are removed. |

### Supplementary Table 2 – Columns created by tidyGWAS

| **tidyGWAS name** | **In output** | **Description** |
| --- | --- | --- |
| CHR | Always | Chromosome |
| POS_37 | Always | Genomic position on GRCh37 |
| POS_38 | Always | Genomic position on GRCh38 |
| RSID | Always | rsID in dbSNP 155 |
| EffectAllele | Always | Allele corresponding to effect |
| OtherAllele | Always | Non effect allele |
| REF_38 | Always | Reference allele on GRCh38 |
| REF_37 | Always | Reference allele on GRCh37 |
| rowid | Always | ID mapping each row to original file |
| multi_allelic | Always | Flags multi-allelic variants |
| Indel | Always | Flags insertions/deletions |
| B | If present in input* | Effect size (beta for linear regression and ln(OR) for logistic regression) |
| Z | If present in input* | Z-score |
| SE | If present in input* | Standard error of effect size |
| EAF | If present in input* | Allele frequency of EffectAllele |
| INFO | If present in input* | Imputation score |
| P | If present in input* | P-value |
| CaseN | If present in input* | Number of cases |
| ControlN | If present in input* | Number of controls |
| N | If present in input* | Total sample size |
| EffectiveN | If CaseN and ControlN in input | Effective sample size |
| Discrep_freq | If flag_discrep_freq is used | Input allele frequencies with absolute difference of >= 0.2 compared to specific reference frequency. |

### **Some columns can be computed or imputed based on the presence of other columns. For example, if B and SE is present, but not Z, tidyGWAS will compute Z.*

| Download directly from GWAS catalog or URL | Yes | No |
| --- | --- | --- |
| Position on both GRCh37 and GRCh38 | Yes | No. MungeSumstats returns information on either GRCh38 or GRCh37. |
| Multi-allelics and Indels | Detected and harmonised with dbSNP. | Multi-allelics are filtered by default.  Indels are excluded from checks related to dbSNP |
| Allele frequencies from reference population | Yes | No |
| Meta-analysis on output | Yes | No |
| Heritability, Genetic correlations, Partitioned heritability | Yes | No |
| Data harmonisation on columns. For example, ensuring SE > 0. | Yes | Yes |
| Imputation of missing columns | Yes,  B,Z,SE, P, N | Yes,  B,Z,SE, P, N |
| Customiseable filters on columns (for example INFO, N, allele frequency) | on EAF | Yes, INFO, N, EAF, multi-allelics, ambiguous SNPs. |
| VCF output | No | Yes |

### Supplementary Table 3 – tidyGWAS compared to MungeSumstats

### *Key differences between MungeSumstats and tidyGWAS*

###
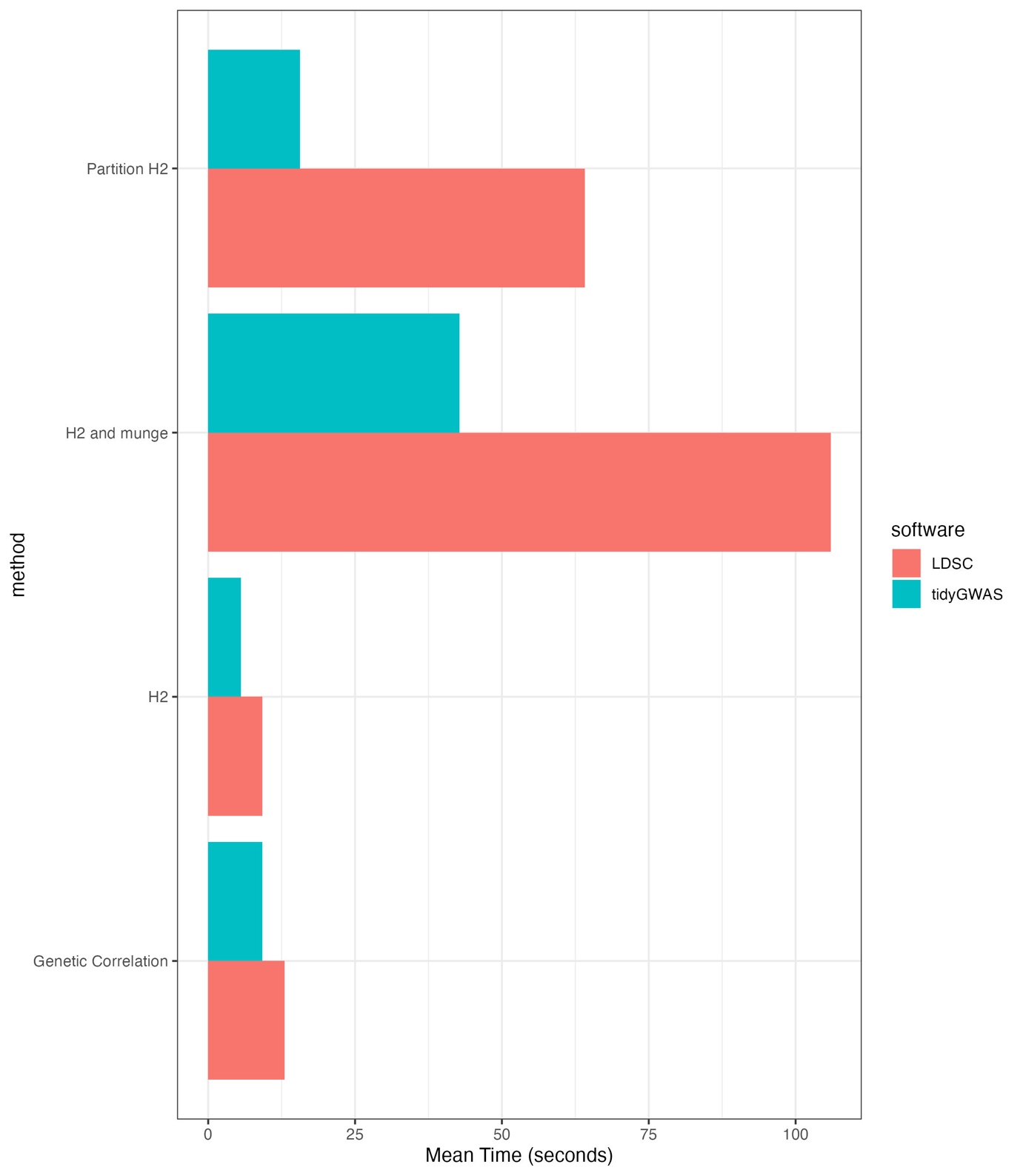
Supplementary Figure 1 – Comparison of ldsR and LDSC

*Performance of tidyGWAS::ldsc_h2, tidyGWAS::ldsc_rg and tidyGWAS::partition_h2(), compared to ldsc.py –h2 and –rg. Partitioned heritability was benchmarked using the 53 baseline annotations*^1^ **h2 and munge refers to running both munge_sumstats.py and ldsc.py.*

1. Finucane, H. K. *et al.* Partitioning heritability by functional annotation using genome-wide association summary statistics. *Nat Genet* **47**, 1228–1235 (2015).
